# Health care provider's experience and perspective of cervical cancer screening in Singapore: A qualitative study

**DOI:** 10.3389/fpubh.2022.853453

**Published:** 2022-07-26

**Authors:** Brandon Wen Bing Chua, Pearlyn Neo, Viva Yan Ma, Li Min Lim, Joseph Soon Yau Ng, Hwee Lin Wee

**Affiliations:** ^1^Saw Swee Hock School of Public Health, National University of Singapore, Singapore, Singapore; ^2^Health Economics and Outcomes Research Center of Excellence (Greater Asia), Becton, Dickinson and Company, Singapore, Singapore; ^3^Strategic Access, Public Affairs, Becton, Dickinson and Company, Singapore, Singapore; ^4^Division of Gynaecologic Oncology, Department of Obstetrics and Gynecology, National University Hospital, Singapore, Singapore; ^5^Department of Pharmacy, Faculty of Science, National University of Singapore, Singapore, Singapore

**Keywords:** cervical cancer screening, Singapore, health care providers, self-sampling, HPV extended genotyping

## Abstract

**Background:**

In Singapore, the current cervical cancer screening (CCS) coverage rate of 48% falls below the national target of 70%. Health care providers (HCPs) play a critical role in promoting CCS uptake. However, there is limited understanding of the perspectives of HCPs regarding CCS. Hence, we aimed to understand the challenges encountered by HCPs delivering CCS in different care settings in the Singapore health system. We also aimed to explore perspectives on newer features of CCS such as self-sampling and HPV genotyping.

**Methods:**

Physicians, nurses, program administrators and laboratory technicians involved with CCS were invited for a one-on-one semi-structured interview conducted over Zoom between May to August 2021. The interviews were transcribed and analyzed using thematic analysis.

**Results:**

Eighteen HCPs from 12 institutions were interviewed. Most participants were women (61.1%) and worked in public health institutions (72.2%). For factors influencing CCS, nine key themes were identified and organized into four categories: (1) patient factors, (2) HCP factors, (3) health system factors and (4) health promotion factors. Key themes commonly highlighted by study participants were related to patients' preferences and acceptance for screening, the processes of delivering CCS, the national priority for cervical cancer and the effectiveness of existing health promotion efforts. Five key themes were identified for CCS innovations. Self-sampling was viewed favorably to increase CCS uptake, while primary HPV screening with HPV partial genotyping had higher sensitivities to detect pre-cancers and cancers compared to cytology. Extended HPV genotyping beyond HPV16/18 could play an important role in CCS with increasing HPV vaccination coverage, as well as in the management of persistent HPV infection.

**Conclusion:**

In Singapore, HCPs face multiple challenges for CCS in practice. Insights from this study are directly relevant to, and useful for developing policies around national CCS programs and treatment guidelines.

## Introduction

Worldwide, cervical cancer affects over 500,000 women annually despite being one of the most preventable cancers (1). Since 2020, the World Health Organization (WHO) has announced a global call to eliminate cervical cancer as a public health problem, by maintaining the disease incidence below 4 per 100,000 women in all countries (2). This is possible by achieving the 90-70-90 target in each country by 2030: (1) 90% of girls fully vaccinated against the human papillomavirus (HPV) by the age of 15, (2) 70% of women screened with high-performance test by the age of 35 and 45, and (3) 90% of women identified with cervical disease receive treatment (2).

Since 2004, the national cervical cancer screening (CCS) program was launched in Singapore to target women aged 25–69 years old for screening (3) Women enrolled in the program are invited to undergo subsidized CCS with a pap smear once every 3 years at government-funded polyclinics, or private general practitioners (GPs) approved under the community health assist scheme (CHAS) (4). Under the CHAS, Singapore citizens can enjoy government subsidies when seeking care for acute and chronic conditions at private GPs (5). In 2019, the national screening guideline was updated and recommended five-yearly HPV test as a screening strategy with HPV genotyping, where HPV16 and HPV18 are individually identified among the 14 high-risk genotypes (HPV partial genotyping) (6). Patients positive for HPV16/18 are referred for colposcopy while those with 12 other high-risk HPV genotypes are managed similarly based on reflex cytology results (6). In addition, school-based HPV vaccination programs have been implemented with a high coverage of 93% for girls aged 12 to 13 years old (7).

Cervical cancer was estimated to cost the Singapore health system $SG57.62 million over a 25-year period beginning in 2008 (8). It remains the 11^th^ most common cancer among women in Singapore, with an age-standardized incidence of 6.87 per 100,000 women (9). While the incidence and survival rate of cervical cancer have been steady in recent years (10), the proportion of late stage cervical cancers have increased (10, 11). This is likely due to the low coverage of CCS, which falls below the national target of 70% (12). In the 2019 population health survey, 48.2% of women surveyed had done a pap smear within the past 3 years, despite 88.5% of them knowing about the purpose of pap smears (13).

Our published literature review identified the factors influencing the uptake of CCS among women in Singapore, which include the lack of time, cost of screening, embarrassment, fear and poor knowledge of screening (14). However, there is limited understanding of the challenges encountered by health care providers (HCPs) delivering CCS. Only one of 14 studies explored the perspectives of CCS HCPs caring for low-income residents in Singapore (15). While the study provided insights on the primary care characteristics that influence CCS (15), the perspectives of providers from the private healthcare sector and tertiary care settings who account for approximately 45% of all CCS in Singapore (13), were not represented. Further, recent innovations in CCS were not considered in previous studies. These include self-sampling to improve CCS uptake (16), the use of primary HPV screening to improve detection of cervical pre-cancers and cancers (17), as well as HPV genotyping beyond HPV16/18 (HPV extended genotyping) to optimize screening triage strategies (18). Hence, the objective of this study was to gather the experiences of HCPs across the health system in Singapore to improve CCS delivery. In addition, we aimed to explore the perspective and readiness of HCPs toward CCS innovations such as HPV genotyping and self-sampling.

## Materials and methods

### Study design and data collection

We conducted in-depth one-on-one interviews to understand the HCPs' experience and perspective on CCS in Singapore. Physicians, nurses, laboratory technicians and program administrators currently involved with CCS were purposively recruited by email invitation. Other inclusion criteria for study participation were: (1) ability to write and read in English, and (2) comfortable with using video conferencing platforms for study participation. We used the snowball sampling method to recruit participants, starting with HCPs from institutions that offered CCS. Referrals for potential study participants were also requested from each study participant recruited. Informed consent was taken prior to the start of the interview.

Due to coronavirus disease-19 (COVID-19) social restrictions, interviews were conducted over Zoom version 5.7.7 (San Jose, CA) for HCPs who agreed to participate in the study. Open-ended questions were asked on the following topics using a semi-structured interview guide: (1) experience with the current national CCS program and screening guideline, including primary HPV screening with HPV partial genotyping, (2) perspectives on the role of HPV extended genotyping, and (3) perspectives on the acceptability and feasibility of self-sampling. Study participants were also asked to provide suggestions and recommendations, based on their experiences. The interview guide was developed by BWBC, and pre-tested with the study team which has expertise in qualitative research methods (PN), health services research (PN, HLW, JSYN, and LML), gynecologic oncology (JSYN, LML) and cervical cancer screening (VYM, JSYN, and LML). The interview guide is available in the Supplementary Material. All interviews were conducted in English by BWBC, a second-year PhD student in public health with 5 years of clinical experience as a pharmacist in women's and children's health, and 6 years of experience in health economics and health services research. BWBC is also an employee of BD, but the company does not have direct input on the design of the interview guide or the conduct of the interviews. All participants had no professional relationship with the interviewer (BWBC). The interviews were conducted between 31 May 2021 to 14 August 2021 with durations ranging between 20 and 60 min. Field notes were taken for one participant who did not agree to have the interview recorded. The remaining interviews were audio-recorded and transcribed verbatim by BWBC. The quality and accuracy of the interview transcripts were further verified by PN. Study participants were given a code and pseudonym to ensure confidentiality and anonymity. Recruitment of study participants continued until data saturation was achieved, defined as the point at which no new themes emerged. A total of 23 potential study participants were invited, 18 of whom agreed to participate in the study. During the consent taking process, a SG$20 reimbursement was offered to all study participants for the time spent. Fifteen participants declined the reimbursement while three accepted the offer.

The anonymized interview transcripts and field notes were stored and analyzed in Dedoose version 9.0.17 (Los Angeles, CA). The six-step Braun and Clark process of inductive thematic analysis was adopted (19), which allowed relevant themes and categories of analysis to emerge from the transcribed interviews. Both semantic and latent approaches were taken to derive codes and themes from the data, without a pre-existing framework. First, familiarization with dataset was done by BWBC, by reading the data at least twice within Dedoose. Next, initial codes were generated systematically across the dataset. Subsequently, the data interpretation was conducted jointly by BWBC, VYM, LML, JSYN, and HLW to minimize bias, blind spots and errors. Patterns within the codes were reviewed and collated into initial themes. Lastly, the initial themes were further refined by re-examining the coherence of codes within each theme. Quotations from participants were also provided to support with our study findings. Recommendations provided by study participants were further organized according to the Chronic Care Model, a framework for improving care delivery while focusing on patient-centered care (20, 21). The framework consists of six elements: (1) health system, (2) community, (3) self-management support, (4) delivery system design, (5) decision support, and (6) clinical information system (20, 21).

### Ethical considerations

The study design, interview guide and quantum of reimbursement were approved by the National University of Singapore Institutional Review Board (NUS-IRB-2021-125).

## Results

A total of 18 HCPs were interviewed from 12 institutions, 11 of which provided CCS services. Majority of the participants were women (61.1%) and worked in public health institutions (72.2%). Half of the study participants had 10 or more years of experience related to CCS. Full demographic details of the study participants are summarized in Table 1.

**Table 1:** Demographics of study participants (*n* = 18).

**Demographics**	**Number of participants**	**%**
**Age group (years)**		
<30	4	22.2%
30–39	4	22.2%
40–49	5	27.8%
≥50	5	27.8%
**Gender**		
Female	11	61.1%
Male	7	38.9%
**Professional expertise**		
General practice/family medicine	7	38.9%
Obstetrics and gynecology	7	38.9%
Others^*^	4	22.2%
**Sector of work**		
Private	5	27.8%
Public	13	72.2%
**Healthcare sector**		
Primary	8	44.4%
Tertiary	9	50.0%
Others	1	5.6%
**Years of experience with cervical cancer screening**		
<10	9	50.0%
10 to 20	3	16.7%
>20	6	33.3%

* Includes nursing, pathology, screening program administration and laboratory services.

**Figure 1:**
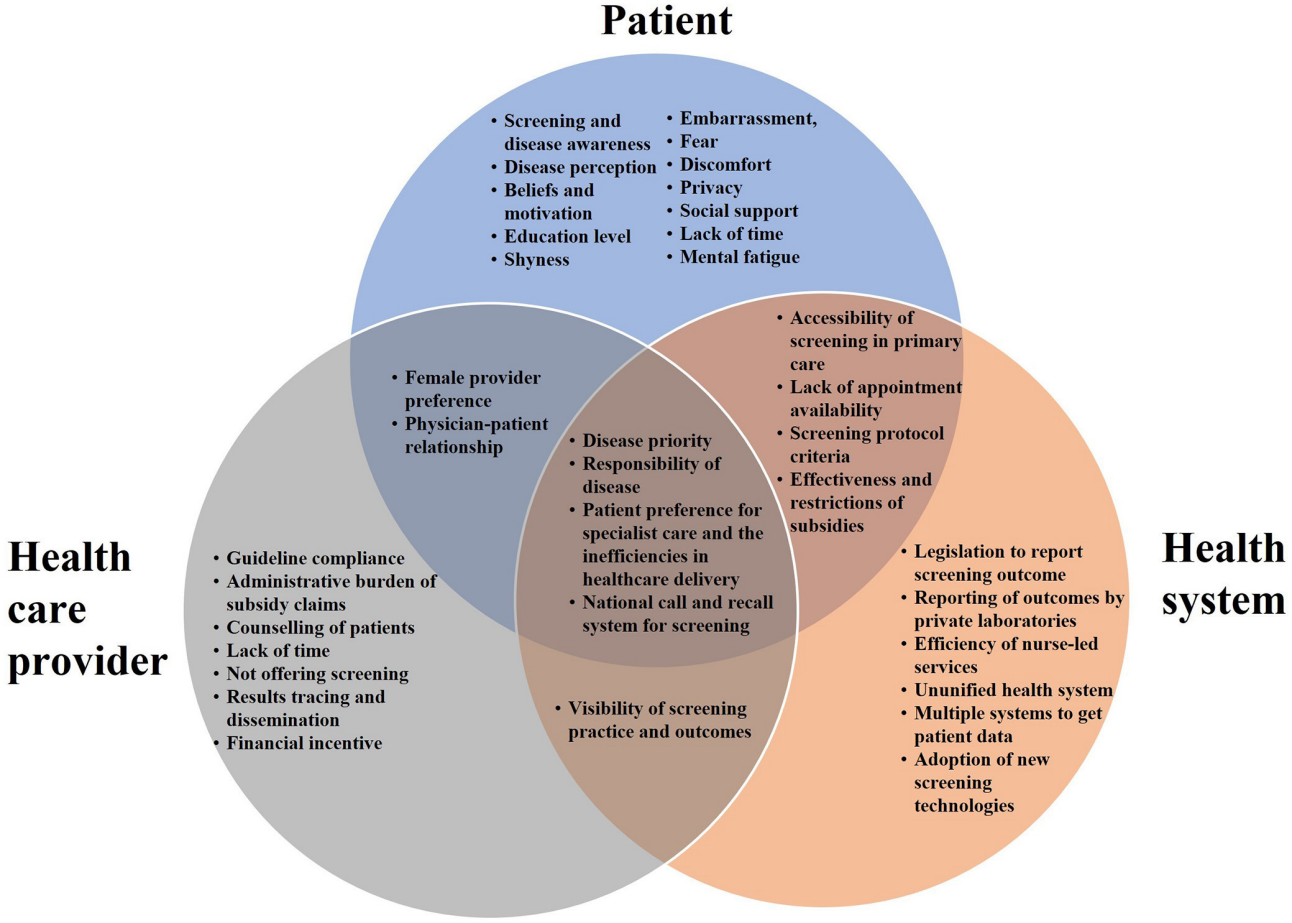
Relationship between factors influencing cervical cancer screening in Singapore.

Table 2 summarizes the nine key themes identified for factors influencing CCS, which were organized into four categories: (1) patient factors, (2) HCP factors, (3) health system factors, and (4) health promotion factors. The relationships between patient, HCP and health system factors are illustrated in Figure 1. The four sub-themes with influences on patients, HCPs and the health system include: (1) disease priority, (2) responsibility for disease, (3) patient preferences for screening and its associated health system inefficiency, and (4) national call and recall system. Five key themes on CCS innovations such as HPV genotyping and self-sampling were identified and summarized in Table 3. Recommendations provided by study participants are shown in Table 4.

**Table 2:** Health care provider's perspectives on factors influencing cervical cancer screening in Singapore.

**Patient factors**	**Health care provider factors**	**Health system factors**	**Health promotion factors**
**Awareness, perception, belief, and motivation toward screening** • Poor awareness of disease and screening • Disease perception • Beliefs and motivation to screen **Preference and acceptance for screening** • Preference for female HCPs in primary care • Preference for specialist care • Lower acceptance to screening in primary care due to mental fatigue from other comorbidities • Differing health priorities with increasing age • Higher acceptance to screening in tertiary care when seen for other gynecological conditions • Discomfort • Privacy • Embarrassment • Fear of results • Shyness **Others** • Low education level and health literacy • Social support • Lack of time for screening	**Time and priority** • Lack of time to discuss screening • Lower disease priority compared to other chronic diseases **Practice of screening** • Providers may not strictly follow national screening guidelines • Financial incentives available for GPs to conduct screening • GPs not offering screening • Inadequate counseling for patients • Female chaperone required for male HCPs • Heavy reliance on HCPs to initiate screening conversation, without the support of systematic reminders • Relationship with patients facilitates screening discussion **Post-screening procedures** • Manual process of tracing and disseminating test results • Administrative burden of subsidy claims among solo-practice GPs • Challenge in discharging patients from tertiary care to primary care for subsequent screening	**National disease priority and organized screening program supported by legislation** • Lower national priority for cervical cancer • Ununified health system with multiple information technology systems to obtain patient information • Lack of national call and recall system • Limited visibility of screening practices, coverage, and outcomes • Limited involvement of private laboratories to report screening results • Lack of legislation to mandate reporting of screening outcomes • Slow national implementation of new screening technologies **Resource allocation in primary and tertiary care setting** • High accessibility of screening services that are helmed at primary care level • Higher efficiency with nurse-led services in polyclinics • Limited availability of appointment slots for screening in polyclinics • Strict screening criterion with number of days post-menstruation in polyclinics • Inefficient resource allocation for screening in tertiary care compared to primary care **Subsidies for cost of screening** • Restriction of screening subsidies • Effectiveness of subsidies in influencing screening uptake	**Effectiveness and delivery of health promotion** • Limited effectiveness in raising awareness compared to other diseases • Lack of age differentiated health promotion • Limitations in delivery of existing health promotion materials

GP, general practitioner; HCP, Health care provider.

**Table 3:** Health care provider's perspectives on different modalities of HPV genotyping and self-sampling for cervical cancer screening.

**HPV genotyping**		
Primary HPV with HPV partial genotyping^*^compared to cytology	**HPV extended genotyping^&^-Utility and value**	**HPV extended genotyping^&^- Considerations for advancement**
• Higher sensitivity to detect precancer • Patient preference for HPV screening based on cost, familiarity with test and screening interval • Increase in cervical biopsy and reduction in cytology workload in laboratories • High efficiencies of workflow to conduct reflex cytology with positive HPV results	• Existing clinical guidelines do not provide recommendations for the management of non-HPV16/18 genotypes • Distinguish non-HPV16/18 genotypes that pose high risk to cervical cancer • Support management of persistent HPV infection • Establish local prevalence of non-HPV16/18 genotypes to guide triaging strategies • Need for monitoring HPV genotypes with changes in HPV epidemiological profile due to vaccination	• Increase evidence awareness on risk of non-HPV16/18 genotypes to guide patient management • Translating existing evidence to clinical practice • Balancing cost and clinical benefits with additional information on HPV genotypes • Clear guidelines on patient management based on HPV extended genotypes
**Self-sampling**		
**Utility and value**	**Implementation considerations and challenges**	
• Higher screening uptake by addressing patient barriers • Increased accessibility to screening • Patient empowerment • Time saving for cervical cancer screening consultations	• Patients' motivations for screening uptake • Lower acceptance among older women • Benefit may be limited for a country with high accessibility to healthcare • Workflow for the distribution of self-sampling kits, labeling and submission of sample • Potential waste of self-sampling kits, if not utilized • Additional cost of screening due to self-sampling kits • Missing other medical conditions without physician examination • Certain age groups and individuals with physical limitations may not be able to perform self-sampling • Challenge of identifying target group • Education on self-sampling procedure • Reliability of patient samples and the need for repeat tests	

* HPV partial genotyping: HPV16 and HPV18 reported individually, with 12 other high-risk HPV genotypes reported as a pooled result

& HPV extended genotyping: Additional genotypes reported individually besides HPV16 and HPV18.

**Table 4:** Recommendations for cervical cancer screening according to the Chronic Care Model framework.

**Recommendations for cervical cancer screening**	**Elements of the chronic care model**					
	**Health system**	**Community**	**Self-management support**	**Delivery system design**	**Decision support**	**Clinical information system**
Promote a culture through digital health or social media, where preventative health is prioritized	✓	✓	✓			
Develop dedicated preventive service packages and train more health care providers for screening in primary care settings	✓			✓		
Shorten patient pathway between diagnosis and referrals for treatment	✓			✓	✓	✓
Mandate organized screening program with national screening registry	✓			✓	✓	✓
Monitor non-HPV16/18 genotypes and implement relevant clinical management pathways	✓				✓	
Explore self-sampling to increase access to screening	✓	✓	✓	✓	✓	

### Patient factors

#### Awareness, perception, belief and motivation toward screening

Study participants described the lack of patient awareness of cervical cancer and screening as a key factor influencing screening uptake in Singapore. Although many women have heard about pap smears, misconceptions about cervical cancer and screening persist. These include the causes of cervical cancer, as well as the purpose and frequency of screening. In addition, barriers to screening are compounded by a poor understanding of the natural history of HPV infection, and consequent anxiety over possible relational fidelity. Often, CCS was deemed to be unnecessary by patients owing to good health, older age, and the lack of sexual activity.

“*Those who, maybe, they come in with a cancer, and then if we ask them, and they said that they have never been for a pap smear before. They*'*ll probably say that, you know, “I've been well all this while and never felt a need to go for regular screening”. They don't understand, or they don't accept the concept of preventative health maintenance, so they say, ‘as long as I don't, I feel well, I don't need to get myself checked up.”’* ID11.

“*They will say that ‘I so old already I do not need la, yeah don't need to check’, or because of their age, they say that they have not have any sexual activities for very long already and they think that it is not necessary.”* ID08.

“*People confuse HPV with other STDs (sexually transmitted diseases), that's one of the other reasons. A lot of women also don't want to come, okay, because when they have positive HPV test, the first thing that they think about is my husband or my partner is sleeping with so many people and that's why I'm having HPV positive … When you Google, HPV is a sexually transmitted disease and they confuse HPV with chlamydia, they confuse HPV with gonorrhea … And all these chlamydia, gonorrhea you need contact tracing, you need antibiotics, you need all that. You don't need this for HPV.”* ID02.

#### Preference and acceptance for screening

The screening preferences of patients also influenced screening practices. There is a strong preference for female HCPs when screening was conducted by GPs. This preference was described to be less significant in the tertiary care settings where female nurses are readily available, and in the polyclinics where screening services are led by female nurses.

“*In the hospital setting, because we have nurses to chaperone. But, if you're in a primary care setting and you're a GP, then you know that you always need to have a chaperone with you when you do vaginal examination and they don't all the time, have a female chaperone next to them.”* ID13.

Besides that, there is a preference to be screened by specialist physicians in the tertiary care setting, where greater assurance and lesser discomfort would be experienced by patients. Patient refusal of screening appeared to be low in the tertiary care setting, especially when patients were also consulting for other gynecological conditions.

“*The patients feel more comfortable in the tertiary care setting number one, because we all gynecologist, we know what we're doing. And number two, because you know, we know that anatomy better, I suppose, and we are more careful. We don't cause pain and we know how to interpret the results properly la, and it's a lot of it about counseling and how comfortable the patient is to the doctor.”* ID13.

However, patients would prefer to receive CCS in primary care settings when they became aware of the lower costs involved, compared to tertiary care settings. When conversations on CCS were initiated in primary care settings, patient acceptance to CCS could be low owing to mental fatigue from other medical conditions. Further, health priorities can differ with age, where older women may be more accepting toward health screening compared to younger women. Other factors that can affect the preference and acceptance toward screening include embarrassment, fear of results, discomfort, shyness and the lack of privacy.

“*Well, I think when you bring up the cost difference, then yes, a lot of patients do prefer to go to the polyclinic because I think in the polys, they only charge $22 and that would also include the reflex pap smear that had to be done. Whereas, in the hospitals itself, you actually pay a lot more.”* ID15.

“*Like if you are the patient, you know, then you would need to, okay so I need to like work on my diet, do my healthy plate, exercise more, 150 min a week. Then you write this blood pressure diary that the doctor gave me, then ‘wah cancer screen ah I think about it next time’, you know, so I think it's a bit overwhelming like that.”* ID18.

### Health care provider factors

#### Time and disease priority

At the primary care level, there are acute conditions and multiple chronic diseases to discuss with patients before preventative health such as CCS would be considered. The limited time available per patient due to high caseload further challenges the feasibility to discuss CCS with patients.

“*The greatest challenge in terms of initiating conversation lies with the clinician because honestly, we are limited in time. Like if you want to discuss a cervical cancer screening, then, it's going to be at least 1 to 2 min. Like say if your queue is 10 persons. You know, like, you have a well patient, then you're probably not going to touch on preventive health, going to spend very little time on it, because that is not priority right. You need to give like on the principle of justice, treat your patients rather equally well, cannot be let them wait 2 h… Preventive is always like the icing on the cake like you got to make sure that you have the main deal of your consult done for the chronic disease first before you can even find time to talk about preventive care. And now-a-days, vaccination is so much more important in this kind of screening, so screening is really the last of the last, only when you have really got the extra time, then you talk about it or unless the person raises it up. yeah, so I think limitation number one is on the clinician side, management of time.”* ID18.

#### Practice of screening

Cervical cancer screening practices varies across treatment setting, where stricter compliance to national guidelines was observed in public hospitals, polyclinics, and CHAS GPs.

*The first thing, the adoption of the HPV primary screening, I can tell you right now polyclinic and the CHAS GP they will all be doing that. Okay, because they will not get paid incentive if they don't follow whatever that is the government national guidelines. They won't get paid, that's the incentive. But in the private each to their own. I still have colleagues doing co-testing every year, I still have doctors doing HPV every year, I still have doctors just sticking to pap smear.”* ID02.

Despite the availability of financial incentives to conduct CCS, there are GPs who do not offer CCS. Male HCPs would require a female chaperone to conduct screening, who may not be readily available. In addition, counseling on CCS provided by existing HCPs was described to be inadequate.

“*Some doctors do not do screening because they've never done a speculum examination before, especially here, the GPs here don't really do a pap smear. Most of the time, the GP practice here, if they have a female GP, they let the female GP do it. They don't do the paps because they don't want to do a speculum examination. They've probably not done for about 20 years or things like that.”* ID02.

Furthermore, the responsibility to initiate discussion on CCS lies heavily on physicians, because few patients would seek consultation for preventive health matters. A good patient-doctor relationship, especially one between a patient and a female HCP, would facilitate such discussions.

“*The system relies on the provider to be prompted to ask the patient ‘hey have you gone for screening?’ Of course, we can check in the system, I remind them la but they rarely come for the sole purpose of asking for cervical cancer screening. They usually come for some other things, but we look at their age, we look at their risk profile, and then we recommend accordingly. So, they come at age of 30 and we recommend HPV screening and then, when they're 35 they come for something else like URTI (upper respiratory tract infection), the onus is on us to be reminded, to ask them la. So, to me that is a huge barrier”*. ID09.

“*Most women like to have it done by somebody they trust and usually it's their own gender, because it involves a very private part of their life … so you have to always like encourage them, remind them, you know, that it is about loving yourself, you know, and you'll be responsible about that*.” ID05.

#### Post-screening procedures

Among solo-practice GPs not affiliated with larger practice organizations, additional manpower is required to submit subsidy claims.

“*I actually have extra staff just doing all the keying in and all the claims. Claims is another part that, you know, the authorities do not realize it takes us a lot of work.”* ID01.

Besides that, the manual process of tracing and disseminating screening results to patients are tedious. In addition, it can take up to 2 weeks before physicians receive test results from the laboratories.

“*I think the notification of results is a challenge for myself because a lot of our things are being done manually. We trace results, abnormal results, we print it out. The doctors print out the results, bring to the colpo room, the nurses look at the results, and then they generate the letter manually and each letter you have to fold, and you know, you have to send it for mailing.”* ID16.

Further, tertiary care physicians face challenges in discharging patients to the primary care for screening. This may be due to patients' anxiety and preference for specialist care. There is also a risk of losing patients to follow-up for subsequent screenings upon discharge to primary care, as patients would have to schedule their own appointments.

“*They just want to follow-up as 1 yearly gynae check-up, right, there are patients who are anxious. So, this group of patients, yeah, they will be with us almost forever, so, and of course, we will just every year do vaginal examination, and do pap smear every 3 to 5 years like that for them”* ID16.

“*It's hard to discharge patient. I'm having difficulties discharging patients to the primary care or to the polyclinics. Because, then the onus is on the patient to go and make an appointment for cervical screen, as compared to here, I just give them an appointment to come back for the next cervical screen or whatever it may be, you know, so the appointments have already been made, so they don't have to worry about it.”* ID13.

### Health system factors

#### National disease priority and organized screening program supported by legislation

Cervical cancer is a key component of the national screening program in Singapore. However, the national priority for cervical cancer is lower compared to other non-communicable diseases such as diabetes. There was a long delay in the adoption of HPV tests and vaccines in the national program, although these technologies have been available in the market for some time. Besides, the national screening program only involves women from polyclinics and CHAS GPs. Hence it does not provide an accurate real-world coverage of CSS in Singapore. Further, there are challenges in recalling patients for subsequent screenings, as automated reminders are only provided for women enrolled in the national program.

“*How would you know when 5 years is to do your next cervical screen, you know? So that's the difficulty we face, because we don't have an automated system that captures this data and automatically, you know, pushes this information out to the patient to say ‘hey look, you know better come for your cervical screen”’* ID13.

The implementation of a national call and recall system is further challenged by the lack of a systematic capture of the national screening practices and results in Singapore. Up to three electronic sources of information may be accessed during clinic consultations to gather relevant patient information: (1) national electronic health record, (2) health institution patient records, and (3) national screening program records. However, obtaining patient history for CCS remains challenging especially when screening results from private health institutions are not integrated with the national electronic health records. Hence, HCPs from public health institutions rely on verbal reports from patients for screening history.

“*So, for us in Singapore, the problem is that we have a different kind of healthcare system. Some people go to private health screening, people go to a GP, people go to hospitals. So, they're doing screenings here and there based on their own preference, right. We don't have a good system to capture who has done screening, who has not done screening.”* ID16.

“*The private as well, nobody's monitoring how many women get pap smear. Are they doing it properly? Are they doing it as per the current evidence-based guideline? No, we don't know basically because we just do not have the registry or the resources to actually, to know what the practice in Singapore is actually like.”* ID2.

Private central laboratories, which serve the majority of primary care institutions in Singapore, can play an active role in reporting screening outcomes. However, further discussions on their involvement have been impeded by logistical and manpower limitations.

“*So, even the private laboratory, assume the private gynecologist do not report, at least the private laboratory can report right. But a lot of them, actually in our previous discussion, they have mentioned that they want to be helpful la, you know, but again logistically you know, manpower requirements.”* ID10.

Ultimately, there are no legislations to mandate the national reporting of screening outcomes, although similar mandates are legislated for the reporting of cancer, human immunodeficiency virus and COVID-19 diagnosis in Singapore. At the same time, existing personal data protection laws such as the Personal Data Protection Act, may hamper data sharing efforts between institutions.

“*It is very difficult. You see the very first instance is ah, we have this major challenge which has been, struggling over many, many years. There's no legislation to mandate compulsory reporting of the pap smear, at least when we are doing pap smear for our screening method. So, there is no compulsion for anyone who do a cervical cancer screening test be it pap or HPV to report that test you see, so we will never know how many people have got the test. And we'll never know how many people was positive and negative because not everybody is being reported on the test, so this is the biggest challenge over the years, because you don't have that then you don't have a denominator to start with ah. But if you no compulsion to report the result then, how do we know how many are positive? So, that is the biggest challenge. I'm not sure how we can overcome it, you know, sort of getting the government to go to Parliament and table a bill, you know, to do that”* ID10.

#### Resource allocation at primary and tertiary care settings

There is high accessibility of screening services helmed at the primary care setting, where patient contact is higher compared to tertiary care settings. Focusing CCS efforts on primary care settings would allow for more efficient allocation of limited healthcare resources, as it frees up capacity for patients who require more urgent obstetrics and gynecology care in tertiary care settings.

“*I feel that tertiary center should be a place where you get referrals when you have abnormal pap smears, right, it shouldn't be places where. I think sometimes also, we have limited clinic slots. Actually, our clinics, especially in the team clinic they are actually very overwhelmed, having patients on follow-ups for things like you know fibroids, ovarian cyst and all kinds of gynecological problems. They are in fact really very overwhelmed, and I know polyclinics is as equally bad you know. In the team clinics, one session, they see, they book, maybe up to 24 patients. So, we can't be a place where people coming in, for just for pap smear. So, I think we have to limit our resources for people who need let's say surgical, you know, specialized, really special, specialist care, following up on things that can't be followed up in polyclinic. Things like pap smear I think it can be done in polyclinic.”* ID16.

In polyclinics, nurse-led women's health services have brought about operational efficiencies for CCS. The screening procedure and counseling are conducted by nurses, allowing physicians to allocate time for other health priorities. However, the limited availability of appointment slots for these services can contribute to long patient wait times before CCS is conducted. Furthermore, CCS appointments will have to be rescheduled if patients are within seven to 10 days of menstruation.

#### Subsidies for cost of screening

There are generous subsidies available to keep screening costs affordable for patients. However, these are available only to Singapore citizens and permanent residents who are screened in primary care institutions under the national screening program. Screening subsidies are often coupled with health education efforts, both of which can influence higher screening uptake. However, the impact of subsidies may be limited by patient's motivations for screening, since screening uptake remains suboptimal in Singapore.

“*Anything you subsidize people are more willing, but subsidies, usually come with health education if you realize it. So, that's the things you see, because the government announces that I am subsidizing this, then people will see how, why is this they are subsidizing, then it comes, it ties in. If you just say health education ah, alone right, but no subsidies to them right, ‘aiya like that only ah,’ you know. Because the thing is everybody wants something more affordable, more accessible.”* ID5.

### Health promotion factors

#### Effectiveness and delivery of health promotion

The limitations of existing health promotion delivery and effectiveness were described by study participants. Existing health promotion materials are not age-targeted and contain generic information on CCS that may not be effective in promoting screening uptake. Besides, information on CCS is often organized together with screening for other cancers which have a higher age eligibility.

“*You give generic information, okay, generic information on the website, it's not really kind of like make women go ‘oh I need to go for this cervical screen’ that's one. Second, you lump everything into cervical cancer screening plus breast cancer screening plus bowel screening, okay, all the information and these are the screening that you do. Okay, and then you put it in a website, and you put it in a generic website. Now, the problem with that, for bowel and breast cancer, the incidence increases when you're 50 and above, and the screening starts from your 50 and above, and we know from a lot of studies cervical screening prevention needs to start from the very, very young. You cannot lump generic information and campaign of a young group with the older generation*.” ID02.

Traditional media such as brochures may have limited impacts in starting conversations on CCS in the age of social media. While virtual seminars and social media have become attractive alternatives for in-person outreach events, they have limited reach to those who are less tech-savvy and disinterested in cervical cancer.

“*Yes, the current brochures are useless la in today's world. You read all these ah, they don't know what's going on. An online presence, having celebrities, Instagram thing, would be I think quite useful, then young people will talk about it among themselves. 20 somethings 30 somethings 40 somethings you hear about it, they start going. I think social media will be quite important.”* ID01.

### HPV genotyping for cervical cancer screening

#### Primary HPV screening with HPV partial genotyping compared to cytology

Primary HPV screening with HPV partial genotyping was described to have a higher sensitivity to detect pre-cancers and cancers, compared to pap smears. The introduction of HPV tests also saw greater efficiencies in CCS when reflex cytology was conducted for abnormal HPV test results. As a result, an increase in cervical biopsy was observed with a corresponding decrease in cytology workload in the laboratory setting. The HPV test is conducted with the same procedure as pap smears and is widely accepted by patients owing to a longer screening interval. However, adoption of HPV test at the primary care level may be limited due to the lack of familiarity among patients, and the higher cost of HPV tests compared to pap smears.

“*Sometimes it's by choice because after counseling, they'll be offered, HPV screening etc., some of them they, don't want to know about HPV, their HPV status for example, then they opt for the conventional pap. Some women have been undergoing pap smears for like 20 years, 30 years already, so they don't see a need to switch to the new test. They still prefer to do the old test which is cheaper.”* ID12.

#### HPV extended genotyping: Utility, value and considerations for advancement

Evidence to inform HPV screening algorithms based on HPV partial genotyping have been developed from western countries. As the HPV prevalence differs in Asian countries, HPV extended genotyping can allow for the prevalence of other high-risk genotypes to be established to guide triaging strategies. As additional HPV genotypes apart from HPV16/18 are reported individually, high-risk HPV genotypes such as HPV52 and HPV45 could be identified with HPV extended genotyping and treated early as they may pose higher risks for cervical pre-cancers and cancers.

“*It's quite useful because there are a few studies that says that in our local context, compared to 16 and 18, actually we do get quite a few, a lot more of things like type 52. And to me, I felt that was actually quite important to distinguish 52 compared to the other high-risk types and I feel that there might actually, there could be a change in the way that we treat screening or cervical cancers. For example, could we put 52 the same as 16/18 for example if it is positive, do we need to go for colposcopy?”* ID15.

Besides, persistent infection with non-HPV16/18 have been observed, where greater knowledge about these genotypes could support with patient management.

“*They have been persistent every year. You do pap smear, HPV test, is other high risk positive. So, some patients actually asked me a question ‘is this the same strain of HPV?.’ Then I will tell them, ‘I don't know, because they don't report which strains is it’... It may be interesting to know which amount these 12 are more common, which are the one that, you know, more high chance to get, you know, high grade CIN.”* ID16.

Participants mentioned that currently patient management do not differ between non-HPV16/18 genotypes. This is because the current screening guideline only has specific management pathways for HPV16/18, while all other HPV genotypes have similar management recommendations. However, the importance of non-HPV16/18 genotypes may increase in the future due to the epidemiological shifts of HPV genotypes brought about by HPV vaccination. Study participants also expressed a need for more guidance on managing patients detected with non-HPV16/18 genotypes.

“*Evidence is going to increase and increase, that basically some of the non-16/18 have a higher risk as well, that is, may be similar to 18. Maybe their way of management might be different from what we are doing now. Okay but the evidence, and when you talk about clinicians, clinicians*' *opinion very different from lab people opinion, from research opinion and all that, because we clinicians are very practical. Okay, because we clinician, all I want to know is what's the risk? Is my management is going to change? Okay, if currently my management's not going to change, I will continue.”* ID 02.

“*As of now, we do not have different pathways of triaging the different genotypes, so that's something that may become important in future, especially when patients are being vaccinated now. So, with the vaccination, you may see a decrease in, for example, the 16 and 18 genotypes, and but other subtypes which may not be covered within the vaccine spectrum may seem, may become more important in future, so it would be good to start collating that kind of information, as to what are the other types of HPV genotypes that are prevalent in the population in Singapore and see whether the future we will see a rise in this kind of cases and also, it may change the morphology of the cases that we are seeing actually. I mean, I don't know if there's concrete evidence to prove that, but people think that maybe with the other genotypes the type of morphological manifestation may show some changes*.” ID04.

More importantly, there is a need to assess and seek to strike a balance between costs and clinical benefits with information provided by HPV extended genotyping.

“*Of course, any form of triaging, any form of added information is always useful. But again, you must always also understand, yeah, if you have the information you need to have some protocol and guidelines to work upon it, you know. It's no point, having information and not doing anything about it. So, it's a balance la, I think. You know, it's good to have information, but you also need the data and the research to drive whether or not you are going to act upon this added information right because, if not, you sort of, the key thing about medicine is never do something which is not going to change your management. It doesn't make sense right just adding on to healthcare costs, and good for research, but not really good for clinical practice.”* ID13.

### Self-sampling for cervical cancer screening

#### Utility and value of self-sampling

Self-sampling was described as an attractive solution to increase CCS uptake as it would address patient barriers related to fear, embarrassment, privacy, and the lack of female HCP. In addition, self-sampling can serve to empower patients to take charge of their health.

“*It will increase coverage. When people are, actually in Asians, actually there are some of them quite shy to come to clinic because your private parts will be exposed, so definitely it will be something that they can do at the comfort of your own home with their privacy with our own, you know, sufficient privacy.”* ID16.

Access to CCS can be improved with self-sampling, as test-kits can be distributed at various locations including pharmacies and vending machines in the community. Further, health institutions may benefit from reduced consultation time spent on CCS with the implementation of self-sampling.

“*With self-sampling, you can actually just make it available everywhere. You can have it as a vending machine, you can have your online, you can have it in the clinic. You can have it in the toilet, busy women's toilet, you can have it even in all the shops. You just pick it up, go to the toilet, just do it, and then just send it back to them, you know. There's a lot of ways to do it. It's just that, as I said before, you can have all these accesses. The main thing is whether the woman wants to do it or not.”* ID02.

#### Implementation considerations and challenges of self-sampling

Acceptance among older women to self-sampling may be limited, owing to resistance to change, lack of familiarity with female hygiene products, and patients' acceptance of paternalism in healthcare. Patients' acceptance toward the self-sampling procedure may also be limited, as suggested by the observation of patients' resistance toward the administration of intra-vaginal medications. In addition, the use of self-sampling test-kits may increase overall CCS costs which may discourage screening uptake.

“*I feel that maybe it would be more targeting, in the younger population who are a bit more savvy, who are more open to this, but I have doubts about, for example, the older population who still prefer the traditional methods of basically seeing a doctor and doing this and they may find it difficult to actually self-collect these samples. It could also be possible because they're not used to different types of menstrual hygiene products, for example, they've always been using a pad. Whereas, the younger generation they've been using things like tampons or the menstrual cups and a bit more familiar with the anatomy below*.” ID15.

The potential resource waste of self-sampling was highlighted by study participants, with parallel comparisons made to mail-out fecal immunochemical test (FIT) kits in Singapore. This is because the success of self-sampling is subject to patients' motivation for screening. Furthermore, a seamless patient pathway would be necessary to prevent any healthcare errors. This includes obtaining self-sampling test kits, receiving education on adequate test procedures, submitting of samples, receiving of test results and subsequent referrals for treatment.

“*They have to know where they are swabbing la, and also, at the same time, they must send out their samples promptly, if they like FIT test when they send by mail like that la yeah. And then also, yeah so, I believe that FIT test also got people who take back the kit, but they don't do anything. They just leave the kits there. So likewise, if this is being brought back to women, I mean, it's given to those women, they also can also put there also”* ID08.

Confidence level on the reliability of self-collected patient samples were mixed among study participants. While some participants have highlighted the equivalence of patient and HCP collected samples based on published studies, others have highlighted the possibility of inadequate samples arising even from HCP obtained samples. Hence, the need to conduct a repeat test for patient obtained samples were mixed.

#### Recommendations for cervical cancer screening in Singapore

Table 4 summarizes the recommendations made by study participants, organized according to the Chronic Care Model framework. The recommendations involved all six elements of framework, where health system was the most common element identified, followed by delivery system design and decision support.

Firstly, a culture prioritizing preventive health could be promoted through digital health or social media. Within the primary care setting, dedicated preventive health service packages could be developed for screening and vaccination, and more HCPs could be trained to provide CCS.

“*In polyclinics, we should have a dedicated service for vaccination and screening, so patients on top of seeing doctors, should also like when they're being triaged, they should go through this service where someone who is hired for the job go through all the age-appropriate screening that they could qualify for and make sure that they are on track… Just someone called a health advocate, for example, who is given a one-day training about, at what age people should go for what kind of screening, what kind of vaccination and then this person's job is just to make sure everyone who goes through polyclinic that day is up to date in terms of their screening and vaccination.”* ID9.

Next, an organized screening program with a national screening registry could be mandated, while patient pathways between diagnosis and referral for treatment could be shortened.

“*There needs to be a national registry for it to work because some women may hop from one place to another … It doesn't really matter where you go and screen, as long as screen right, so you want to be able to capture this data in the national registry, so that all health care providers can have access to this. And then, these women can be followed up wherever, I don't care which hospital they go to, you know, as long as they are followed up and they are screened for. So, I think that should be the aim, and the registry allows you to track for that, allows you to send reminders to the patients wherever they are in Singapore, you know just a reminder to go for screening when they're supposed to go for screening.”* ID4.

Besides that, monitoring of extended HPV genotypes could be implemented together with relevant clinical management pathways. Lastly, self-sampling could be explored to increase access to CCS.

## Discussion

Cervical cancer remains a significant clinical diagnosis in Singapore, and effective treatment alone would not suffice for cervical cancer prevention. While systematic age-appropriate HPV vaccination have been adopted (7), a similar adoption have not been observed for CCS. In this study, we have interviewed a wide range of HCPs across the Singapore health system to understand the impact of patient, provider, health system and health promotion factors on CCS. For the first time, we are able to investigate the factors affecting CCS holistically. We identified the downside of not having an organized screening program with a call and recall capacity, challenges for expanding CCS under current clinical settings and patient preference for screening settings. In particular, a lower acceptance to screening was observed in primary care settings compared to tertiary care settings, which has not been reported in studies from other high-income countries (22–24) This may be driven by preferences for specialist care among women, as well as the primary care physicians' lack of time to discuss CCS in light of other health priorities. Unlike previous research in Singapore (14), we also explored perspectives on recent CCS innovations, such as self-sampling and HPV genotyping, and their role in clinical practice. In addition, we found that with increasing HPV vaccination rate, HPV extended genotyping could play an important role under the shifting epidemiological paradigm of HPV genotypes, as well as the management of persistent HPV infection. Insights from this study are directly relevant to, and useful for developing policies around national CCS programs and treatment guidelines.

Key challenges faced by HCPs in Singapore suggest that investments in infrastructures for CCS, such as a comprehensive national call-recall type registry, may be necessary. With a central registry to track age-eligible patients, send automatic screening reminders, and track responses, CCS uptake could be improved while reinforcing the importance of age-appropriate CCS as a national priority. This shifts the heavy reliance on physicians to monitor and initiate CCS conversations, to one that is shared between the health system, patient, and HCP. At the same time, the concerns of tertiary care physicians of losing patients to follow-up for subsequent screening in the primary care settings can be minimized. However, substantial financial resources and legislative support are required for the implementation of an organized screening program backed with a national screening registry. For example, Australia, which is poised as the first country to eliminate cervical cancer (25), had allocated AU$220 million to build and manage the National Cancer Screening Register over a 5 year period (26). It is therefore crucial to evaluate the long-term cost-effectiveness of such infrastructures in Singapore. With the decreasing incidence and mortality of cervical cancer in Singapore (11), gathering these financial and legislative support can be challenging. At the same time, care delivery and capacities in primary care settings for CCS needs to be reconsidered for such infrastructures to be effective. This is because current primary care capacities may already be overwhelmed with the management of chronic diseases as seen from this study, resulting in the lack of time among HCPs to discuss CCS with patients. Nevertheless, such infrastructures should still be considered as a national priority in Singapore as benefits can also be reaped for the national monitoring of other cancers without an organized, call and recall screening approach, such as breast and colorectal cancer. Systematic screening is in line with the Singapore government's current emphasis on prevention and early detection as a cost-effective approach to national health improvements (27). Hence, a national registry approach will help improve compliance and provide actionable data for policy decision support.

Next, health promotion approaches toward CCS could be reconsidered, as the lack of awareness of cervical cancer and screening continues to be a key challenge for improving CCS uptake in Singapore. This highlights the lack of a deeper understanding of the importance of CCS, as screening coverage remains suboptimal despite an increasing awareness of pap smears among Singaporean women (13). Other possible reasons include limitations in health literacy and access to health information (28). With the advent of COVID-19, significant changes in the delivery and consumption of health information have been observed with the accelerated adoption of digital and mobile health technologies in Singapore. Majority of Singaporeans have begun using online sources of information for health promotion and disease prevention (29). Further, the Singapore government has utilized social media and WhatsApp extensively to provide daily updates on COVID-19 and to combat misinformation (29, 30). These have significant implications and represent significant opportunities for health promotion delivery, as mobile health interventions involving patient education and reminders for screening can increase CCS uptake by 88% (31). Further, social media can shape health behavior (32), and harnessing this power to increase consciousness around evidence-based cost-effective approaches to personal health is important. In addition, national digital health platforms, such as HealthHub, has been established since 2016 to enable greater accessibility to health information (33). These should be leveraged together with the digital and mobile health technology momentum generated by COVID-19 to complement existing efforts on cervical cancer prevention.

Recent innovations in CCS that empower patients and provide more personalized care can also be explored to improve CCS delivery in Singapore. Globally, self-sampling can improve CCS uptake by up to 2-folds compared to standard screening provided by HCPs (34). It provides a patient-centered approach to CCS by addressing multiple barriers to screening faced by women in clinical settings (35). These include embarrassment, the lack of time and the absence of female HCPs (14, 36, 37). In this study, self-sampling was acknowledged as a favorable initiative to increase CCS uptake and access in Singapore, while reducing consultation time spent in clinics. This may address health system inefficiencies stemming from patients' preference for screening by specialist physicians, as CCS can be conducted in the comforts of home. Implementation considerations for patient pathways and wastage were also highlighted in this study, suggesting the need for a deeper understanding on patient's preferences for CCS service delivery with self-sampling. Ongoing studies are evaluating the acceptability and feasibility of self-sampling among women from a public health institution in Singapore (38). Coupled with the insights gathered from this study on service implementation, national patient pathways for CCS with self-sampling can be designed and implemented subsequently in the national screening program.

The use of HPV tests for CCS can also influence care delivery for patients, by improving the detection of pre-cancers and cancers (17). Since 2019, Singapore has adopted HPV genotyping as a primary screening method, recommended by the Society for Colposcopy & Cervical Pathology of Singapore (6). In the current study, primary HPV screening with HPV partial genotyping was found to be widely accepted by patients, and has a higher sensitivity over pap smears to detect pre-cancers and cancers. When HPV extended genotyping beyond HPV16/18 was considered, better risk stratification of patients could be made based on HPV genotype specific risk for cervical intraepithelial neoplasia (CIN) or invasive cancer. This is significant as recent evidence suggests that the risk for CIN grade 3 or more severe diagnoses (CIN3+) is stratified across a wide range for the 12 other high-risk HPV genotypes (39). Hence, reporting non-HPV16/18 genotypes as a pooled result would underestimate the HPV genotype specific risk of CIN3+, especially HPV31, HPV58 and HPV33 (39). Further, the value of HPV extended genotyping to monitor persistent same genotype HPV infections was acknowledged in this study. This has significant implications for clinical practice as persistent same genotype infections are associated with higher risk of CIN2+ (40). Taken together, these suggest that risk-based CCS guidelines that considers HPV genotypes beyond HPV16/18 have a growing role in CCS programs. While further evaluations are required to ensure the cost-effective implementation of these algorithms in Singapore, an evaluation in the US has shown that they can be cost-effective in comparison to HPV16/18-only based screening algorithms (41).

Our study has its limitations. Firstly, this study adopted a qualitative study approach, which alone, may have limited generalizability of the Singapore HCPs' perspective. However, the qualitative approach allowed us to generate rich insights on the perception of HCPs for CCS which can inform future quantitative studies. Next, purposive sampling was adopted for the recruitment of study participants which may lead to selection bias. Besides that, representatives from private laboratories were not included in this study due to challenges in study recruitment brought about by COVID-19. Private laboratories support the majority of primary care institutions in Singapore and would provide additional insights on the local CCS program. Nevertheless, some of these challenges have been raised by study participants who had prior exchanges with private laboratories.

## Conclusion

In Singapore, there is a rising trend of late-stage cervical cancer, possibly due to suboptimal CCS coverage. Multiple challenges for CCS are faced by HCPs across the health system. Patient, HCP, health system and health promotion level gaps in CCS were highlighted in this study, along with considerations for self-sampling and HPV genotyping. These may inform health policy makers to develop interventions to improve CCS service delivery in Singapore. Future studies can explore patients' preferences for CCS, as well as the role of HPV extended genotyping and self-sampling in the national screening program. With a lower prioritization for cervical cancer owing to decreasing incidence and mortality rates, further investments for improvements in CCS service delivery may be a challenge. However, this should not hamper the momentum of eliminating cervical cancer, one of the most preventable cancers in the world.

## Data availability statement

The original contributions presented in the study are included in the article/Supplementary material, further inquiries can be directed to the corresponding author/s.

## Ethics statement

The studies involving human participants were reviewed and approved by National University of Singapore Institutional Review Board (NUS-IRB-2021-125). The patients/participants provided their written informed consent to participate in this study.

## Author contributions

BWBC was involved with conceiving the study, developing the methods, collecting, and analyzing the data, drafting and revising of the manuscript. PN was involved with developing the methods and drafting of the manuscript. LML and JSYN were involved with developing the methods, as well as drafting and revising the manuscript. VYM and HLW were involved with conceiving the study, developing the methods, supervising BC, and revising the manuscript. All authors approved the final manuscript.

## Funding

BWBC is a Doctor of Philosophy candidate in the Industrial Postgraduate Programme with the National University of Singapore. Under the Industrial Postgraduate Programme, tuition fee, allowance and research funding support were provided by the Economic Develop Board (Singapore). The study reimbursements were provided by Becton Dickinson.

## Conflict of interest

The authors declare that the research was conducted by a PhD student (BWBC) who is a staff of a medical device company. The participants were informed of the interviewer's (BWBC) work background prior to providing consent to participate.

## Publisher's note

All claims expressed in this article are solely those of the authors and do not necessarily represent those of their affiliated organizations, or those of the publisher, the editors and the reviewers. Any product that may be evaluated in this article, or claim that may be made by its manufacturer, is not guaranteed or endorsed by the publisher.
