# Supplementary material for: Health care provider's experience and perspective of cervical cancer screening in Singapore: A qualitative study
Source: Front Public Health. 2022 Jul 26;10:853453. doi: 10.3389/fpubh.2022.853453 (PMC9360748; doi:10.3389/fpubh.2022.853453)
Supplement: Supplementary file 1 [file Table_1.DOCX]

Supplementary Material

# Interview guide for physicians and nurses

| **Interview questions** | **Themes/sub-themes** |
| --- | --- |
| **Introductory question**  Can you tell me more about yourself?  You have mentioned earlier that you have had X years of experience with cervical cancer screening program in Singapore.   Can you begin by telling us what do you do/about your day-to-day task with regards cervical cancer screening? | **Demographics**   \| Age group in 2021 (years old) \| <30 / 30-39/ 40-49/ 50-59/ >60 \| \| --- \| --- \| \| Gender \| male/ female/ prefer not to say \| \| Location of work \| hospital/ general practice clinic/ polyclinic/ others \| \| Sector of work \| public/ private \| \| Years involved in cervical cancer screening \|  \|   **Role in cervical cancer screening**  - Do you conduct screening yourself? Do you refer your patients to other clinics for screening? - Do you think there is a preference among women to receive cervical cancer screening in a private or public health institute? Why? |
| **National screening program and guideline**  What are your thoughts and perspective of the current national screening program and guideline? (e.g. current understanding of what it involves, screening criteria and tests available)  Prompt if unsure: briefly, the current national screening program involves personalized letters to eligible women to attend screening from polyclinic and public hospitals every 3-5 yearly depending on the screening type. Clinical guidelines for cervical cancer screening and management have been updated in 2019. | **National guidelines on cervical cancer screening and management**  - What are your thoughts on the screening guidelines?  - How clear are the screening criteria specified in the guidelines?  (to providers and patients) **National cervical screening program**  - How are women eligible for screening monitored for screening attendance and follow-up care?  - Are there available initiatives to minimize the number of women lost to follow-up?  **Access**  - Subsidized screening is available at CHAS GPs. How effective is this initiative in increasing screening uptake?  **National disease priority**  - How does cervical cancer rank as a disease priority in Singapore  - What are your views regarding the health promotion efforts for cervical cancer screening?  **Strengths and weaknesses**  - Overall, what are the strengths and weaknesses of the existing cervical cancer screening program and guideline? |
| **Challenges faced as a provider**  What are some of the challenges faced as a provider in your daily practice in the field of cervical cancer screening? | **Priority of condition in practice**  - How often have you come across patients who query about cervical cancer screening in your practice - How often do you initiate conversations about cervical cancer screening for eligible patients?  **Operational requirements for screening**  - How do you find the process of determining eligible patients for screening?  (e.g. obtaining accurate screening history from patients)  - How do you find the process of obtaining a sample for cervical cancer screening?  (e.g. equipment used, procedure, space required, female preference, not enough manpower)  **Incentives**  - Are there adequate incentives for screening promotion as a provider?  **Facilitators**  - How can providers be motivated to initiate conversations on cervical cancer screening with patients? |
| **Technology adoption**  What are your key considerations for adopting new technologies in your day-to-day work?  What about the national screening program? | **Primary HPV**  - Primary HPV has been introduced in 2019 as a screening modality. What are your views on primary HPV in relation to pap smears? and co-testing?  - How has this guidance affected your day-to-day work since its introduction? in the future?  **HPV genotyping beyond HPV16/18 (HPV extended genotyping)**  - There are 14 high risk HPV genotypes that cause cervical cancer. The vaccine in Singapore covers for 16 and 18, but emerging research tells us that other genotypes can be risky as well. New assays are available can identify additional genotypes beyond HPV16/18, giving you more information to manage the patient. What do you think about what I have just shared? - How would such emerging assays for HPV tests impact the practice of cervical cancer screening in Singapore?  - If you were to incorporate extended genotyping or genotyping beyond HPV16/18 in practice, how would you do it? If not, why not?  **Self-sampling**  - Self-sampling involves patients obtaining their owns samples for HPV test compared to receiving an inspection from a healthcare worker.  - How would self-sampling impact the practice of cervical cancer in Singapore?  (acceptability among patients/providers, implementation challenges) |
| **Barriers among women**  Do you feel screening uptake for cervical cancer among women are within expectations in Singapore? | Why do you think that women do not attend cervical cancer screening in Singapore?  Possible prompters:  **Social support**  - To what extent would family/ friends play a part in screening uptake?  **Fear, embarrassment, social stigma**  - Is cervical cancer screening a taboo topic at present?  **Inconvenience**  - Do you think there is a preference for screening in the public institutions or private institutions? (estimate a proportion)  **Cost**  - Would cost be a main consideration for screening?  **Disease perception and knowledge**  - How much do you think women in Singapore know about cervical cancer? - How do you think women perceive cervical cancer compared to other diseases?  **Target groups**  - Are there any age groups to target screening efforts? Why?  - Are there religious groups to target for screening efforts? Why?  **Facilitators**  - How can cervical cancer uptake be improved among women? |
| **Concluding question/ suggestions/ recommendations**  Before we conclude this interview, is there anything else you would like to add? | - If there are 1 or 2 changes you could make to change the way cervical cancer is screened in Singapore, what would you do? What recommendations would you make? |

# Interview guide for laboratory technicians

| **Interview questions** | **Themes/sub-themes** |
| --- | --- |
| **Introductory question**  Can you tell me more about yourself?  You have mentioned earlier that you have had X years of experience with cervical cancer screening program in Singapore. Can you begin by telling us what do you do/about your day-to-day task with regards cervical cancer screening? | **Demographics**   \| Age group in 2021 (years old) \| <30 / 30-40/ 40-50/ 50-60/ >60 \| \| --- \| --- \| \| Gender \| male/ female/ prefer not to say \| \| Location of work \| hospital/ general practice clinic/ polyclinic/ others \| \| Sector of work \| public/ private \| \| Years involved with cervical cancer screening \|  \|   **Role in cervical cancer screening**  - Are you involved in receiving cervical cancer screening samples from both private and public institutions?  - What is the estimated distribution of private/public and why? |
| **National screening program and guideline**  What are your thoughts and perspective of the current national screening program and guideline?  Prompt if unsure: briefly, the current national screening program involves personalized letters to eligible women to attend screening from polyclinic and public hospitals every 3-5 yearly depending on the screening type. Clinical guidelines for cervical cancer screening and management have been updated in 2019. | **National cervical screening program and national guidelines on cervical cancer screening and management**  - How do the guidelines and screening program influence your day-to-day work? |
| **Challenges faced as a provider**  What are some of the challenges faced as a provider in your daily practice in the field of cervical cancer screening? | **Operational requirements for screening**  - How do you find the process of preparing and analyzing samples for cervical cancer screening?  (e.g. equipment used, procedure/turnover time, space required, manpower)  - How does the processing of cervical cancer screening samples contribute to the overall operational requirements of the laboratory?  **Incentives**  - Are there adequate incentives for screening promotion as a provider? |
| **Technology adoption**  What are your key considerations for adopting new technologies in your day-to-day work?  What about the national screening program? | **Primary HPV**  - Primary HPV has been introduced in 2019 as a screening modality. What are your views on primary HPV in relation to pap smears? and co-testing?  - How has this guidance affected your day-to-day work since its introduction? in the future?  **HPV genotyping beyond HPV16/18 (HPV extended genotyping)** - There are 14 high risk HPV genotypes that cause cervical cancer. The vaccine in Singapore covers for 16 and 18, but emerging research tells us that other genotypes can be risky as well. New assays are available can identify additional genotypes beyond HPV16/18, giving you more information to manage the patient. What do you think about what I have just shared? - How would such emerging assays for HPV tests impact the practice of cervical cancer screening in Singapore?  - If you were to incorporate extended genotyping or genotyping beyond HPV16/18 in practice, how would you do it? If not, why not?  **Self-sampling**  - Self-sampling involves patients obtaining their owns samples for HPV test compared to receiving an inspection from a healthcare worker.  - How would self-sampling impact the practice of cervical cancer in Singapore?  (acceptability among patients/providers, implementation challenges) |
| **Concluding question/ suggestions/ recommendations**  Before we conclude this interview, is there anything else you would like to add? | - If there are 1 or 2 changes you could make to change the way cervical cancer is screened in Singapore, what would you do? What recommendations would you make? |

# Interview guide for program administrators

| **Interview questions** | **Themes/sub-themes** |
| --- | --- |
| **Introductory question**  Can you tell me more about yourself?  You have mentioned earlier that you have had X years of experience with cervical cancer screening program in Singapore. Can you begin by telling us what do you do/about your day-to-day task with regards cervical cancer screening? | **Demographics**   \| Age group in 2021 (years old) \| <30 / 30-40/ 40-50/ 50-60/ >60 \| \| --- \| --- \| \| Gender \| male/ female/ prefer not to say \| \| Location of work \| hospital/ general practice clinic/ polyclinic/ others \| \| Sector of work \| public/ private \| \| Years involved in cervical cancer screening \|  \|   **Role in cervical cancer screening**  - How are you involved with monitoring of the screening program  - Do you think there is a preference among women to receive cervical cancer screening in a private or public health institute? Why? |
| **National screening program and guideline**  What are your thoughts and perspective of the current national screening program and guideline? (e.g. current understanding of what it involves, screening criteria and tests available)  Prompt if unsure: briefly, the current national screening program involves personalized letters to eligible women to attend screening from polyclinic and public hospitals every 3-5 yearly depending on the screening type. Clinical guidelines for cervical cancer screening and management have been updated in 2019. | **National guidelines on cervical cancer screening and management**  - What are your thoughts on the screening guidelines?  - How clear are the screening criteria specified in the guidelines?  (to providers and patients) **National cervical screening program**  - How are women eligible for screening monitored for screening attendance and follow-up care?  - Are there available initiatives to minimize the number of women lost to follow-up?  **Access**  - Subsidized screening is available at CHAS GPs. How effective is this initiative in increasing screening uptake?  **National disease priority**  - How does cervical cancer rank as a disease priority in Singapore  - What are your views regarding the health promotion efforts for cervical cancer screening?  **Strengths and weaknesses**  - Overall, what are the strengths and weaknesses of the existing cervical cancer screening program and guideline? |
| **Challenges faced as a provider**  What are some of the challenges faced as a program administrator in your daily practice in the field of cervical cancer screening? | **Operational requirements for screening**  - How do you find the process of documenting screening results for patients in Singapore? (private setting vs public setting)  - How do you find the process of notifying eligible patients for screening?  **Incentives**  - Are there adequate incentives for screening promotion for healthcare providers?  **Facilitators**  - How can healthcare providers be motivated to initiate conversations on cervical cancer screening with patients? |
| **Barriers among women**  Do you feel screening uptake for cervical cancer among women are within expectations in Singapore? | Why do you think that women do not attend cervical cancer screening in Singapore?  Possible prompters:  **Social support**  - To what extent would family/ friends play a part in screening uptake?  **Fear, embarrassment, social stigma**  - Is cervical cancer screening a taboo topic at present?  **Inconvenience**  - Do you think there is a preference for screening in the public institutions or private institutions? (estimate a proportion)  **Cost**  - Would cost be a main consideration for screening?  **Disease perception and knowledge**  - How much do you think women in Singapore know about cervical cancer? - How do you think women perceive cervical cancer compared to other diseases?  **Target groups**  - Are there any age groups to target screening efforts? Why?  - Are there religious groups to target for screening efforts? Why?  **Facilitators**  - How can cervical cancer uptake be improved among women? |
| **Technology adoption**  What are your key considerations for adopting new technologies in your day-to-day work?  What about the national screening program? | **Primary HPV**  - Primary HPV has been introduced in 2019 as a screening modality. What are your views on primary HPV in relation to pap smears? and co-testing?  - How has this guidance affected your day-to-day work since its introduction? in the future?  **HPV genotyping beyond HPV16/18 (HPV extended genotyping)**  - There are 14 high risk HPV genotypes that cause cervical cancer. The vaccine in Singapore covers for 16 and 18, but emerging research tells us that other genotypes can be risky as well. New assays are available can identify additional genotypes beyond HPV16/18, giving you more information to manage the patient. What do you think about what I have just shared? - How would such emerging assays for HPV tests impact the practice of cervical cancer screening in Singapore?  - If you were to incorporate extended genotyping or genotyping beyond HPV16/18 in practice, how would you do it? If not, why not?  **Self-sampling**  - Self-sampling involves patients obtaining their owns samples for HPV test compared to receiving an inspection from a healthcare worker.  - How would self-sampling impact the practice of cervical cancer in Singapore?  (acceptability among patients/providers, implementation challenges) |
| **Concluding question / suggestions/ recommendations**  Before we conclude this interview, is there anything else you would like to add? | - If there are 1 or 2 changes you could make to change the way cervical cancer is screened in Singapore, what would you do? What recommendations would you make? |
